# Determination of deleterious single-nucleotide polymorphisms of human *LYZ C* gene: an in silico study

**DOI:** 10.1186/s43141-022-00383-8

**Published:** 2022-07-01

**Authors:** Harini Venkata Subbiah, Polani Ramesh Babu, Usha Subbiah

**Affiliations:** 1 Human Genetics Research Centre, Sree Balaji Dental College & Hospital, Bharath Institute of Higher Education and Research, Chennai, Tamil Nadu India; 2 Center for Materials Engineering and Regenerative Medicine, Bharath Institute of Higher Education and Research, Chennai, Tamil Nadu India

**Keywords:** In silico, Polymorphism, Missense, Lysozyme

## Abstract

**Background:**

Single-nucleotide polymorphisms (SNPs) have a crucial function in affecting the susceptibility of individuals to diseases and also determine how an individual responds to different treatment options. The present study aimed to predict and characterize deleterious missense nonsynonymous SNPs (nsSNPs) of lysozyme C (*LYZ C*) gene using different computational methods. Lyz C is an important antimicrobial peptide capable of damaging the peptidoglycan layer of bacteria leading to osmotic shock and cell death. The nsSNPs were first analyzed by SIFT and PolyPhen v2 tools. The nsSNPs predicted as deleterious were then assessed by other in silico tools — SNAP, PROVEAN, PhD-SNP, and SNPs & GO. These SNPs were further examined by I-Mutant 3.0 and ConSurf. GeneMANIA and STRING tools were used to study the interaction network of the *LYZ C* gene. NetSurfP 2.0 was used to predict the secondary structure of Lyz C protein. The impact of variations on the structural characteristics of the protein was studied by HOPE analysis. The structures of wild type and variants were predicted by SWISS-MODEL web server, and energy minimization was carried out using XenoPlot software. TM-align tool was used to predict root-mean-square deviation (RMSD) and template modeling (TM) scores.

**Results:**

Eight missense nsSNPs (T88N, I74T, F75I, D67H, W82R, D85H, R80C, and R116S) were found to be potentially deleterious. I-Mutant 3.0 determined that the variants decreased the stability of the protein. ConSurf predicted rs121913547, rs121913549, and rs387906536 nsSNPs to be conserved. Interaction network tools showed that LYZ C protein interacted with lactoferrin (LTF). HOPE tool analyzed differences in physicochemical properties between wild type and variants. TM-align tool predicted the alignment score, and the protein folding was found to be identical. PyMOL was used to visualize the superimposition of variants over wild type.

**Conclusion:**

This study ascertained the deleterious missense nsSNPs of the *LYZ C* gene and could be used in further experimental analysis. These high-risk nsSNPs could be used as molecular targets for diagnostic and therapeutic interventions.

## Background

Single-nucleotide polymorphism (SNP) is a variation at a single position in the genetic sequence and one of the common sources of sequence alterations in humans which are present in greater than 1% of the population [1]. SNPs can occur in the gene coding regions producing a change in the amino acid (nonsynonymous), or can be silent without causing a change in the amino acid (synonymous), or can occur in the noncoding regions (5′untranslated region (UTR), 3′UTR, and introns).

Missense nonsynonymous SNPs (nsSNPs) can produce a variation in the amino acid sequence and have the ability to alter the structure and function of a protein, thereby affecting disease pathogenesis and progression in individuals. SNPs can also affect gene expression by influencing promoter activity, conformation and stability of messenger RNA (mRNA), and translational efficiency regulating the susceptibility of individuals to diseases, drug metabolism, and genomic evolution [2].

Deleterious SNPs of various genes have been identified using in silico methods, and some of them include lactoferrin (*LTF*) [3], mitochondrial tumor suppressor 1 (*MTUS1*) [4], guanosine triphosphate cyclohydrolase 1 (*GCH1*) [5], gap junction protein alpha 3 (*GJA3*) [6], beta-defensin 1 (*DEFB1*) [7], B-cell lymphoma/leukemia 11A (*BCL11A*) [8], and apoptosis protease-activating factor 1 (*APAF1*) [9]. Observing and recording DNA polymorphisms in different genes and populations can help in developing personalized medicines [10].

Antimicrobial peptides (AMPs) are principal constituents of the innate immune system and have suppressive effects on bacteria, fungi, viruses, and parasites [11]. Lysozyme C (LYZ C) is an important AMP secreted in body secretions such as milk, tears, and saliva. LYZ C cleaves β (1, 4) glycosidic bond linkage between N-acetyl muramic acid and N-acetyl glucosamine of the peptidoglycan layer of bacteria, thereby causing loss of membrane integrity and leading to osmotic lysis of bacteria [12]. As it is one of the important AMPs, polymorphisms in the *LYZ C* gene can reduce its antimicrobial potential and increase the susceptibility to infections.

Not all SNPs identified are deleterious, and it is important to distinguish deleterious SNPs from neutral SNPs. The high number of SNPs makes it difficult to carry out experiments in the laboratory to find out the importance and biological contribution of each SNP. However, computational tools can be used to initially filter potentially damaging SNPs that might affect susceptibility to diseases and drug metabolism before further laboratory investigations. This study analyzed missense nsSNPs of *LYZ C* gene and the effect of variants on the protein’s three-dimensional structure and function.

## Methods

### Retrieval of nsSNPs

The National Center for Biotechnology Information (NCBI)-SNP database (https://www.ncbi.nlm.nih.gov/snp/) was used to retrieve the SNPs of the *LYZ* C gene (accessed on 3 September 2021). Only the missense nsSNPs of the *LYZ* gene were retrieved from the database as the nucleotide change results in an altered codon that codes for a different amino acid and potentially impacts the structural and functional features of the protein. The FASTA sequence of LYZ C protein was obtained from the UniProt web server (accession number is P61626, accessed on 3 September 2021).

### Prediction of deleterious missense nsSNPs

Several web servers were used to distinguish deleterious nsSNPs from neutral ones. First, the missense nsSNPs obtained from the NCBI-SNP database were submitted to SIFT (Sorting Intolerant from Tolerant; http://sift.bii.a-star.edu.sg/) and PolyPhen v2 (Polymorphism Phenotyping v2; http: //genetics.bwh. harvard.edu/pph2/) tools. SIFT uses a query sequence and builds a multiple sequence alignment and based on position-specific information predicts tolerated and deleterious substitutions [13]. A substitution in the protein sequence that is conserved in the alignment will be scored as intolerant to most changes, and a poorly conserved substitution will be scored as tolerating [14]. SIFT analyzes the occurrence of a new amino acid at a position, and the normalized score ranges from 0 to 1. A score between 0 and 0.05 is determined to be deleterious, and the value above the cutoff of 0.05 is considered tolerant [15]. PolyPhen v2 predicts the consequence of amino acid variants by doing multiple sequence alignments, phylogenetic predictions, and analyzing structural features [16]. The result of the PolyPhen v2 is a numerical score varying from 0.0 (benign) to 1.0 (damaging) and a prediction showing the substitution as probably damaging, possibly damaging, or benign. SIFT and PolyPhen are able to predict 90% of deleterious SNPs and are the representatives of the empirical rule-based method which uses a set of empirical rules based on sequence homology, evolutionary conservation, and structural features characterizing a particular variant.

To increase the accuracy of prediction, the nsSNPs that were found to be deleterious by both SIFT and PolyPhen were subjected to the following tools. SNAP (Screening for Non-Acceptable Polymorphisms, https://rostlab.org/services/snap/) provides a sequence-based prediction and incorporates evolutionarily conserved information, that is how a residue is conserved within the sequence families and also uses other predicted information such as secondary structure and solvent accessibility and analyzes whether a SNP has any effect on function (non-neutral) or no effect (neutral) [17]. PROVEAN (Protein Variation Effect Analyzer (http://provean.jcvi.org)) uses a region-based alignment score that measures the effect of amino acid variation not only at the position of interest but also takes into account the alignment of neighborhood flanking sequences for determining the consequence of the variant on the functional aspect of the protein [18]. PROVEAN score cutoff of ≤ −2.5 suggests that the amino acid variant has a deleterious effect, whereas the variant having a score > −2.5 is regarded to have a neutral effect on the function of the protein. PhD-SNP (Predictor of Human Deleterious — Single-Nucleotide Polymorphism, http://snps.biofold.org/phd-snp/phd-snp.html) tool is based on support vector machines (SVMs) that use protein sequence and predicts whether a nsSNP is associated with a genetic disease in humans [19]. SNPs & GO (GO-Gene Ontology, http://snps-and-go.biocomp.unibo.it/snps-and-go/) uses evolutionary information and function as encoded in the GO sequence-associated terms and predicts whether the variation has any effect on the gene functionality [20].

### Prediction of protein stability change

I-Mutant 3.0 tool (http://gpcr.biocomp.unibo.it/cgi/predictors/I-Mutant3.0/I-Mutant3.0.cgi) was employed to predict how a single-point variation affects the thermodynamic stability of the protein. It is focused on the difference in free energy changes (Delta Delta G (DDG)) between the wild-type and variant proteins [21]. The output of I-Mutant 3.0 is a DDG value that is calculated from the protein’s sequence or tertiary structure with the following predictions: DDG < −0.5 kcal/mol is largely unstable, DDG > 0.5kcal/mol is largely stable, or −0.5 ≤ DDG ≤ 0.5 kcal/mol is neutral.

### Evolutionary conservation analysis

ConSurf web server (http://consurf.tau.ac.il/) was used to study the evolutionary conservation of amino acid position in LYZ C protein. ConSurf tool first develops a multiple sequence alignment of the given sequence, constructs a phylogenetic tree, and gives a position-specific conservation score [22]. The score range from 1 to 9, where 1 indicates the variable region, 5 mildly evolving position, and 9 indicates conserved position.

### Gene-gene interaction

Studying the gene interaction network is of prime importance to understand the disease phenomenon. As the genes are interlinked, a mutation in a gene can affect its interacting partners in the network, and therefore, it is important to analyze disease-related genes [23]. GeneMANIA tool (http://www.genemania.org) predicted the gene interaction network of the *LYZ C* gene.

### Protein-protein interaction

Proteins are part of complex molecular mechanisms, and it is important to identify protein-protein interactions to elucidate the function of proteins and their specific roles in the disease process. The protein-protein interaction of LYZ C protein was studied by the STRING (Search Tool for the Retrieval of Interacting Genes) tool (https://string-db.org/) [24].

### Secondary structure prediction

NetSurfP 2.0 (https://services.healthtech.dtu.dk/service.php?NetSurfP-2.0) uses a primary sequence and detects the surface accessibility and secondary structure of a protein [25]. The secondary structure of LYZ C protein was predicted by NetSurf P.

### Variant analysis by HOPE tool

HOPE (Have (y) Our Protein Explained) is an web-based application that analyzes the impacts of point mutations on the structure and function carried out by a protein [26]. It builds homology models and collects information including sequence interpretations from the UniProt database, 3D coordinates of the protein, and develops a detailed report with the characteristics and effects of the mutation in comparison with the wild-type protein.

### Protein modeling and structural analysis

Protein homology modeling was carried out for both wild type and variants using the SWISS-MODEL web server (https://swissmodel.expasy.org/). The quality of the models was examined and analyzed by online servers such as PROCHECK, ERRAT, VERIFY3D, and PROVE (https://saves.mbi.ucla.edu/). Energy minimization was carried out using XenoPlot software with the steepest descent and 1000 steps per structure with a resolution of 10 Å. Amber_94 force field was utilized to minimize the energy of the molecule to a more stable position. The 3D models built for wild type and variants were uploaded in TM-align tool (https://zhanggroup.org/TM-align/) to get root-mean-square deviation (RMSD), align, and template modeling (TM) scores [27]. The tool generates residue-to-residue alignment based on the similarity and gives a TM score which has a value between 0 and 1, where 1 indicates similarity between two structures. Scores below 0.2 correspond to randomly chosen unrelated proteins, while scores above 0.5 assume the same fold in SCOP/CATH. Superimposition of variants over wild type was carried out using PyMOL.

## Results

### nsSNPs retrieval from NCBI-SNP database

The nsSNPs of the *LYZ* C gene were extracted from the NCBI-SNP database. There were a total of 2855 SNPs out of which 44 were synonymous, 105 were missense, 1351 introns, and others. Missense nsSNPs were selected for further analysis as a change in the coding sequence could result in altered protein sequence and hence could affect the protein structure rendering protein nonfunctional and increasing the susceptibility to different diseases. But not all missense substitutions affect protein’s structure and function; some remain neutral without causing significant changes. Hence, it is essential to distinguish deleterious SNPs from neutral ones.

### Prediction of deleterious missense nsSNPs

First, 105 missense nsSNPs were subjected to SIFT tool which showed that 12 nsSNPs were deleterious with a SIFT score less than ≤ 0.05. The nsSNPs were then subjected to the PolyPhen v2 tool. To increase the accuracy of prediction, both SIFT and PolyPhen v2 tool results were taken into consideration. The nsSNPs having SIFT score ≤ 0.05 and PolyPhen v2 score > 0.90 were considered for further investigation. SIFT and PolyPhen v2 tools predicted 8 SNPs to be deleterious and probably damaging, respectively. These 8 SNPs were further submitted to other online tools — SNAP, PROVEAN, PhD-SNP, and SNPs & GO. The results of the in silico tools are presented in Table 1.

**Table 1:** Analysis of deleterious missense nsSNPs by various bioinformatics tools

SNP ID	Amino acid change	SIFT (Score)	PolyPhen (Score)	SNAP	PROVEAN	PHD-SNP	SNPs & GO
rs1800973	T88N	Deleterious (0.01)	PD (0.997)	Effect	Deleterious	Neutral	Neutral
rs121913547	I74T	Deleterious (0.001)	PD (1)	Effect	Deleterious	Disease	Disease
rs121913549	F75I	Deleterious (0.001)	PD(0.925)	Effect	Deleterious	Disease	Disease
rs387906535	D67H	Deleterious (0.026)	PD (0.979)	Effect	Deleterious	Disease	Disease
rs387906536	W82R	Deleterious (0)	PD (1)	Effect	Deleterious	Disease	Disease
rs121913548	D85H	Deleterious (0.02)	PD (0.979)	Effect	Deleterious	Disease	Disease
rs147465274	R80C	Deleterious (0.046)	PD (0.967)	Effect	Deleterious	Disease	Disease
rs200491782	R116S	Deleterious (0.016)	PD (0.994)	Effect	Deleterious	Disease	Neutral

*PD* Probably damaging

### Determination of the effect of missense nsSNPs on Lyz C stability

Advances in different genotyping methods have led to the identification of a significant number of missense variations. The consequence of amino acid substitutions on protein stability will help to predict variations that lead to disease phenotypes [28]. I-Mutant 3.0 was used to analyze whether the variants increased or decreased the stability of the protein or remained neutral. The variants that had a DDG value less than −0.5 decreased the stability of protein and are shown in Table 2.

**Table 2:** Prediction by I-Mutant 3.0

SNP ID	Amino acid change	DDG value (Kcal/mol)	Prediction
rs1800973	T88N	−1.08	Decrease
rs121913547	I74T	−2.72	Decrease
rs121913549	F75I	−0.80	Decrease
rs387906535	D67H	−0.23	Neutral
s387906536	W82R	−1.23	Decrease
rs121913548	D85H	−0.49	Neutral
rs147465274	R80C	−1.10	Decrease
rs200491782	R116S	−1.02	Decrease

### Evolutionary conservation analysis

Slowly evolving sites on the protein molecule are critical for its function, and the ConSurf tool predicts evolutionarily conserved regions in the protein query macromolecule [29]. ConSurf predicted rs121913547 (I74T), rs121913549 (F75I), and rs387906536 (W82R) nsSNPs of LYZ C protein to be conserved. rs121913549 (F75I) and rs387906536 (W82R) had structural and functional importance, respectively. The results of the ConSurf analysis are given in Table 3.

**Table 3:** Prediction of evolutionary conservation by ConSurf

SNP ID	Amino acid change	Prediction (Score)	ConSurf Prediction	Functional/structural
rs1800973	T88N	3	Variable	-
rs121913547	I74T	8	Conserved	-
rs121913549	F75I	9	Conserved	s
rs387906535	D67H	6	Moderately conserved	-
rs387906536	W82R	9	Conserved	f
rs121913548	D85H	5	Moderately conserved	-
rs147465274	R80C	4	Moderately conserved	-
rs200491782	R116S	4	Moderately conserved	-

According to the ConSurf server, “f” — functional residue and “s” — structural residue

### Gene-gene interaction network of LYZ C gene

**Fig. 1:**
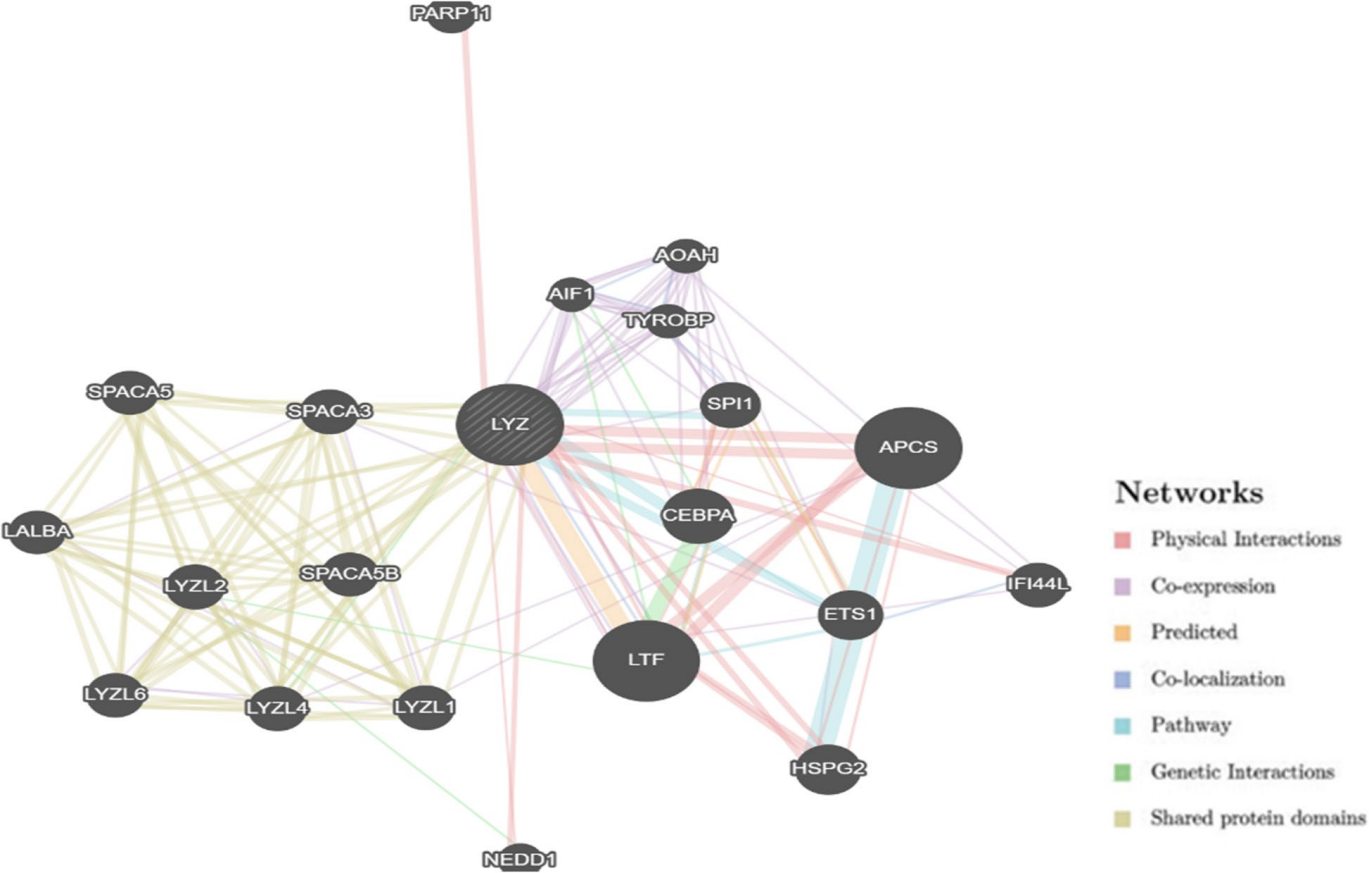
Gene-gene interaction network of *LYZ* C gene predicted by GeneMANIA

GeneMANIA tool predicted that *LYZ C* and lactoferrin (*LTF*) interact with each other. Both are components of innate immunity and form a part of the first line of defense against microbes. *LYZ C* gene has physical interactions with the following genes — amyloid P component, serum (*APCS*), heparin sulfate proteoglycan 2 (*HSPG2*), interferon-induced protein 44 like (*IFI44L*), and poly (ADP-ribose) polymerase family member 11 (*PARP11*). The interaction network of the *LYZ C* gene as predicted by GeneMANIA is shown in Fig. 1.

### Analysis of protein-protein interaction

**Fig. 2:**
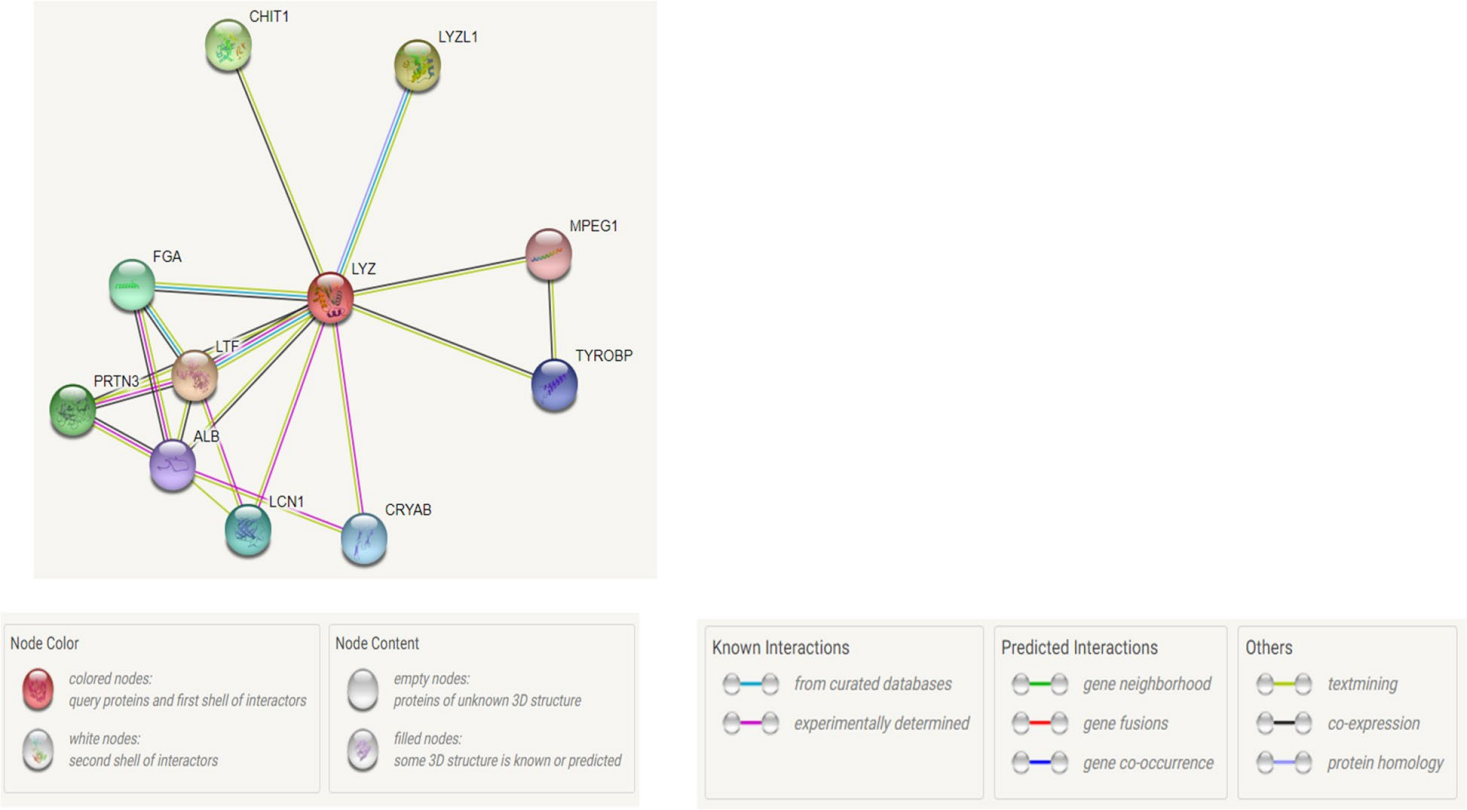
Protein-protein interaction of LYZ C by STRING tool

Proteins function in a coordinated fashion and interact with other proteins to carry out cell signaling and other functions of a cell. Therefore, amino acid variations in a protein can affect other proteins in a network. The functional partners of LYZ as predicted by the STRING tool are LTF, lysozyme-like protein 1(LYZL1), chitotriosidase-1 (CHIT1), myeloblastin serine protease (PRTN3), fibrinogen alpha chain (FGA), lipocalin-1 (LCN1), alpha-crystallin B chain (CRYAB), transmembrane immune signaling adaptor (TYROBP), serum albumin (ALB), and macrophage-expressed gene 1 protein (MPEG1). LTF was found to be the main interacting partner of LYZ with a maximum score of 0.874. Both gene and protein interaction networks predicted that LYZ C interacts with LTF and possibly acts in a synergistic manner against the invading microbes. The protein-protein interaction network of LYZ C protein is given in Fig. 2.

### Secondary structure prediction of LYZ C

**Fig. 3:**
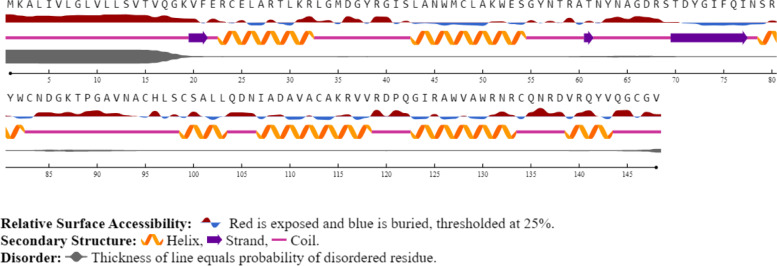
Secondary structure prediction of LYZ C by NerSurfP

Helix and strand formed by the amino acids of LYZ C protein are mapped to their primary sequence and given in Fig. 3.

### Effect of polymorphism by HOPE analysis

HOPE tool analyzed the consequences of variation on LYZ protein’s 3D structure and function by comparing the physicochemical properties between variant and wild-type amino acids. The effect of variation is shown in Table 4.

**Table 4:**
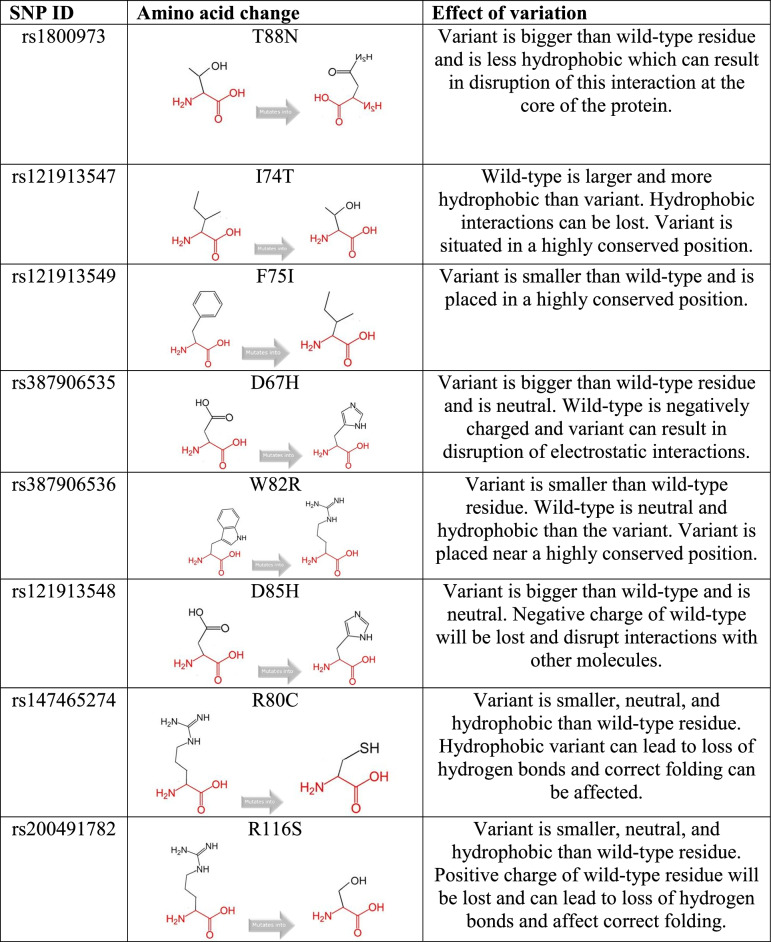
Effect of polymorphism as analyzed by HOPE tool

### Structural analysis and superimposition of variant over wild type

Homology modeling scores of the structures are given in Table 5. The structures were evaluated by different tools and shown in Table 6. TM align is a protein structure comparison tool and does alignment based on structural similarity. RMSD, align score, TM score, and superimposition images are given in Table 7. TM score for the variants and wild types was found to be between 0.5 and 1 indicating that protein folding is identical.

**Table 5:** Homology modeling scores as predicted by SWISS-MODEL server

Name	MolProbity score	Clash score	Ramachandran score	Q mean value
Wild	1.17	0.49	97.71	0.90
T88N	1.17	0.49	97.71	0.91
I74T	1.07	0.49	97.71	0.90
F75I	1.17	0.49	97.71	0.90
D67H	1.17	0.48	97.71	0.90
W82R	1.00	0.00	97.71	0.90
D85H	1.29	0.97	97.71	0.89
R80C	1.17	0.49	97.71	0.90
R116S	1.17	0.49	97.71	0.91

**Table 6:** 3D structure evaluation by different online tools

Name	PROCHECK score	ERRAT quality score	Verify_3D	PROVE_score
Wild	Out of 8 evaluations • 1: Errors • 4: Warning • 3: Pass	98.4	100.00% of amino acids have 3D-1D average score > = 0.2 Pass	Total buried outlier atoms of protein: 1.3%
T88N	Out of 8 evaluations • 1: Errors • 4: Warning • 3: Pass	99.2	100.00% of amino acids have 3D-1D average score > = 0.2 Pass	Total buried outlier atoms of protein: 1.3%
I74T	Out of 8 evaluations • 1: Errors • 4: Warning • 3: Pass	97.6	100.00% of amino acids have 3D-1D average score > = 0.2 Pass	Total buried outlier atoms of protein: 1.8%
F75I	Out of 8 evaluations • 1: Errors • 4: Warning • 3: Pass	97.6	100.00% of amino acids have 3D-1D average score > = 0.2 Pass	Total buried outlier atoms of protein: 1.3%
D67H	Out of 8 evaluations • 1: Errors • 4: Warning • 3: Pass	98.4	100.00% of amino acids have 3D-1D average score > = 0.2 Pass	Total buried outlier atoms of protein: 1.5%
W82R	Out of 8 evaluations • 1: Errors • 4: Warning • 3: Pass	99.2	100.00% of amino acids have 3D-1D average score > = 0.2 Pass	Total buried outlier atoms of protein: 1.3%
D85H	Out of 8 evaluations • 1: Errors • 4: Warning • 3: Pass	99.2	100.00% of amino acids have 3D-1D average score > = 0.2 Pass	Total buried outlier atoms of protein: 1.3%
R80C	Out of 8 evaluations • 1: Errors • 4: Warning • 3: Pass	99.2	100.00% of amino acids have 3D-1D average score > = 0.2 Pass	Total buried outlier atoms of protein: 1.3%
R116S	Out of 8 evaluations • 1: Errors • 4: Warning • 3: Pass	98.4	100.00% of amino acids have 3D-1D average score > = 0.2 Pass	Total buried outlier atoms of protein: 1.3%

**Table 7:**
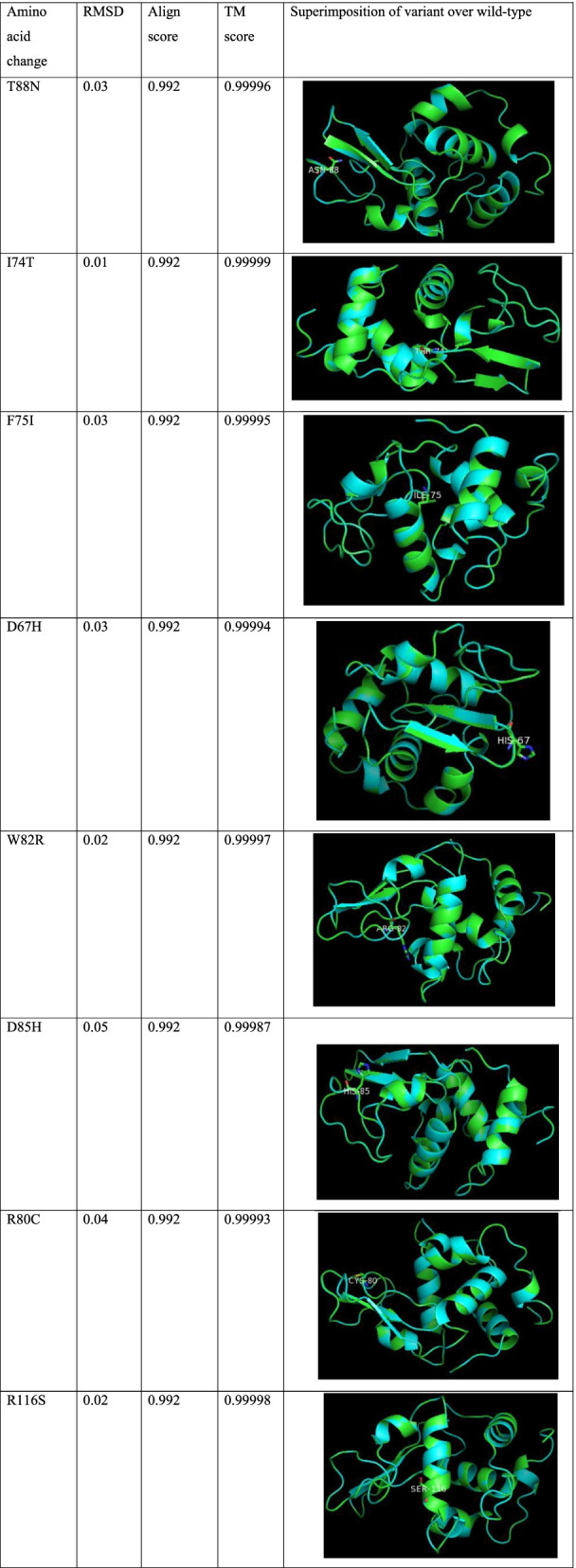
Predictions as given by TM-align tool and superimposition by PyMOL

## Discussion

Identifying biologically relevant SNPs can help in developing SNP-based genetic profile that can be used as genetic screening markers in identifying the risk of individuals to different diseases and help in studying inheritance patterns [30]. Polymorphisms in drug-metabolizing enzymes, drug transporters, and genes that code for drug receptors can lead to inter-individual variations in drug response and influence the development of personalized diets and medicines [10, 30].

The current research work identified the impact of nsSNPs of the *LYZ C* gene on the structural and functional aspects using various in silico tools. First, 105 missense nsSNPs obtained from the NCBI-SNP database were analyzed by SIFT and PolyPhen tools which predicted 8 nsSNPS to be deleterious. These SNPs were then studied by other tools such as SNAP, PROVEAN, Phd-SNP, and SNPs & GO. Then, the effect of these deleterious nsSNPs on protein stability was studied by I-Mutant 3.0 tool which compared the free energy change between wild type and variants. Change of amino acids located in the conserved region produces deleterious effects, and the ConSurf tool was employed to study the phylogenetic conservation of amino acids. ConSurf predicted polymorphisms I74T, F75I, and W82R of LYZ C to be conserved. Gene-gene and protein-protein interaction tools suggested that LTF and LYZ interact and possibly produce a synergistic effect. AMPs such as LTF synergize with LYZ, whereby LTF permeabilizes the outer membrane of gram-negative bacteria and enhances the access of LYZ to the peptidoglycan layer for the effective killing of gram-negative bacteria [31]. LTF also sequesters iron and limits the iron availability to bacteria inhibiting its growth. This interaction enhances host defense. Polymorphisms in LYZ C can affect the other genes and proteins in the interaction network, thereby affecting cell signaling and biological pathways. HOPE tool analyzed the effect of a variation in the native amino acid sequence by studying the physicochemical properties such as substitution between hydrophobic and hydrophilic amino acids, burial or exposure of charged and neutral residues, loss of non-covalent interactions such as hydrogen bonds, electrostatic interactions, and disruption of covalent interactions such as disulfide bonds. TM score provides the topological similarity between variant and wild-type proteins, while the RMSD value provides the average distance between alpha-carbon backbones of the two models. Higher RMSD values predict greater variant structure deviation from wild type. In this study, both the values as predicted by the TM-align tool showed that the protein folding was identical between wild type and variant.

Polymorphisms in host genes can increase or decrease susceptibility to diseases by altering the host’s ability to fight against infections. Mutations can also occur in microbial genomes causing an increase in virulence leading to higher infectivity and transmission rates. The variant of severe acute respiratory syndrome coronavirus 2 (SARS-CoV-2), D614G, was the predominant form which increased the affinity of the virus to bind to human receptor angiotensin-converting enzyme 2 (ACE2) [32]. There were also other variants in circulation that led to changes in the structure of the spike protein which the virus used to bind to human cell receptors more effectively. Influenza viruses more commonly undergo certain phenomena called antigenic drift and antigenic shift [33]. Antigenic drift occurs when there is a point mutation in the genes. A new variant arises when there is a mutation in genes encoding surface proteins hemagglutinin and neuraminidase. The individuals become more susceptible to the new variant. Antigenic shift occurs when there is a reassortment of the segmented genome with another influenza virus changing their surface antigens drastically. For example, in a pig (animal reservoir) infected with both a human strain and an avian strain of influenza, reassortment can result in surface antigens containing a combination of both the strain’s genes. These variants cannot be detected by the immune system. Antigenic shifts can result in pandemics. These are some of the reasons for mutations in microbial genomes specifically in a virus. Polymorphisms in human genes can result in a decreased ability of the immune system to detect these mutations that occur in microbes.

Lysozyme can inhibit virus entry by preventing its binding with cell receptors and thereby virus-mediated cell fusion. As lysozyme is an important AMP, deleterious polymorphisms can affect the structure and hence the function of LYZ C resulting in decreased immunity against infections including viral diseases. In silico tools have been used to predict deleterious missense nsSNPs of the *LYZ C* gene, and these nsSNPs need to be further validated by experimental procedures. Once experimentally demonstrated and validated, these deleterious SNPs could be useful in developing a panel of biomarkers to predict the susceptibility of individuals to different diseases.

## Conclusion

In this study, out of 105 missense nsSNPs of the *LYZ C* gene, 8 nsSNPS were predicted to be deleterious by various bioinformatics tools. The effect of these missense nsSNPs of the *LYZ C* gene on protein structure and function needs to be confirmed by experimental investigations. The use of multiple in silico methods provides cost-effective and rapid screening which could guide further laboratory analyses.

## Data Availability

All data analyzed during this study are included in this article.

## Abbreviations

ACE2: Angiotensin-converting enzyme 2
ALB: Albumin
AMP: Antimicrobial peptide
APCS: Amyloid P component, serum
CHIT1: Chitotriosidase-1
CRYAB: Alpha-crystallin B chain
DDG: Delta Delta G
FGA: Fibrinogen alpha chain
GO: Gene ontology
HOPE: Have (y) Our Protein Explained
HSPG2: Heparan sulfate proteoglycan 2
IFI44L: Interferon-induced protein 44 like
LCN1: Lipocalin-1
LTF: Lactoferrin
LYZ C: Lysozyme C
LYZL1: Lysozyme-like protein 1
MPEG1: Macrophage-expressed gene 1 protein
NCBI: *National Center for Biotechnology Information*
nsSNPs: Nonsynonymous single-nucleotide polymorphisms
PARP11: Poly (ADP-ribose) polymerase family member 11
PhD-SNP: Predictor of human deleterious single-nucleotide polymorphism
PolyPhen: Polymorphism phenotyping
PROVEAN: Protein Variation Effect Analyzer
PRTN3: Myeloblastin serine protease
RMSD: Root-mean-square deviation
SARS-CoV-2: Severe acute respiratory syndrome coronavirus 2
SIFT: Sorting intolerant from tolerant
SNAP: Screening for non-acceptable polymorphisms
SNP: Single-nucleotide polymorphism
SVM: Support vector machine
TM: Template modeling
TYROBP: Transmembrane immune signaling adaptor
UTR: Untranslated regions
